# Correction: Evaluating the long-term strength of GGBFS-blended cement across various water-to-binder and superplasticizer ratios under heating/cooling cycles

**DOI:** 10.1371/journal.pone.0335994

**Published:** 2025-11-03

**Authors:** Bashdar Omer, Najmadeen Mohammed Saeed, Mahmood Hunar Dheyaaldin, Ahmed Salah Jamal, Rawaz Kurda

There are errors in the author affiliations. The correct affiliations are as follows:

Bashdar Omer^1^, Najmadeen Mohammed Saeed^1^, Mahmood Hunar Dheyaaldin^2,3^, Ahmed Salah Jamal^4^, Rawaz Kurda^5^

**1** Civil Engineering Department, University of Raparin, Rania, Iraq, **2** Department of Civil Engineering, Cihan University-Erbil, Erbil, Iraq, **3** Department of Civil Engineering, American University in Dubai, UAE, **4** Civil Engineering Department, Faculty of Engineering, Tishk International University, Erbil, Iraq, **5** Department of Highway and Bridge Engineering, Technical Engineering College, Erbil Polytechnic University, Erbil, Iraq.
