# Supplementary material for: Evaluating the long-term strength of GGBFS-blended cement across various water-to-binder and superplasticizer ratios under heating/cooling cycles
Source: PLoS One. 2025 Jul 24;20(7):e0319923. doi: 10.1371/journal.pone.0319923 (PMC12289012; doi:10.1371/journal.pone.0319923)
Supplement: Attachment — Submitted filename: Plos reviewes comments[1].docx [file pone.0319923.s001.docx]

Reviewer #1: Reviewer comments
1- Abstract: The abstract should be improved and include clear statements about objectives, methodology and findings.

Reply:

The abstract has been altered according to the reviewer’s comment.

2. Relationship to Literature: The paper is bad structured and poorly recent documented, the amount of data is insufficient. It is necessary more recent and appropriate literature. Review more published studies to enhance the background the present study. Recent related references need to be incorporated. Introduction paragraph one needs proper references.

Reply: as the reviewer requested, more recent references have been added to the introduction.

3. Methodology: The methods employed are robust and appropriate but more was needed in order to arrive a more information and results. Rebuild the section (method and experimental (RSM)- results and discussion).

Reply: In order to make the introduction clearer, the first paragraph of section 2 was altered.

4. It is better to provide the images of the specimens prepared for the test.

Reply: please, see Figure 3. The specimens were shown.

5. the paper only describes results and does not discuss findings with other studies.

Reply: The authors agree with the reviewer’s comment and this step is an important parameter to be considered for any research. Nevertheless, after conducting an in-depth literature review, it was revealed that there is little to no data on the evaluation of GGBFS blended cement under different GGBFS replacement contents, w/b ratios, and SP dosages. Thus, we could not make a comparison between the data of this study and the others.

6 . A flowchart should be provided for the work process. The flow chart of the study has to be described in the steps.

Reply: as shown in Figure 3 (Fig 1. A schematic of the experimental process.), In this study, we tried to focus on the work process instead the whole process. Instead of the flowchart, we described the work process of the work as a text (see, the first paragraph of section 2).

7. Why is it based only on the compressive strength (where are the other mechanical strengths)?

Reply: IN this work, we studied 27 different mixes. These specimens were then removed after 24 hours and subjected to water curing for 28, 56, 90, and 365 days. Our objective to mainly focus on the compressive strength. Other tests can be added for future works such as a book.

8. improve the presentability of figures 12a to 12c (badly illustrated)

Reply:

Based on the reviewers’ comment, the figures have been changed and the trend line was added to understand the trend.

Reviewer #2:

The manuscript was crafted in a distinctive way in which the presented experimental results were validated using response surface methodology as well as Anova. Both statistical results reinforced and backed the findings of the author, as expected, that every experimental result should have either statistical, simulation, or machine learning model validation.
The typing error in line 5 under the flowability of result and discussion heading should be corrected from "ADDINg" to "adding."

Reply: Thank you very much for your valuable comment. As the reviewer requested, the word “ADDing” was corrected.

Reviewer #3:

Doesn’t meet the criteria for publication. Infect doesn’t have required elements of a research paper.
A superficial lab test in terms of mixture test (Table 1), use of many undefined acronyms, , inadequate samples, … In terms of innovation doesn’t have any advantage over

<https://iopscience.iop.org/article/10.1088/1755-1315/498/1/012045>,

<https://www.sciencedirect.com/science/article/pii/S2666790823000095>

Reply: Both papers have been considered in the study.

Typically disorganized,

1. Long and vague title

Reply: the tittle has been altered according to the

2. Poor English with massive flaws which must extensively be revised by a native expert

Reply: The English has been checked again and we only found a minor

3. Not critically analyzed relevant works. Simply the gap can be seen in reference list.
4. Lack of any Discussion, solid comparison, limitation, practical and pitfall difficulties

8. Obvious inadequate sample for any generalizing, statistical analysis

Reply: comments 4 and 8 in which they are similar. We decided to reply them jointly. The discussion section was 25 pages. This is way more than any usual paper. We don’t mind to discuss more but the limitation of the journal gaudiness may not allow that.

5. Outdated references with almost belong to more than two decades ago

Reply: The list of the references has been updated

6. Unhighlighted research gaps with at least 3 years gaps!!!! WONDERED no work after 2021????? See the given example above.

Reply: The relevant studies have been updated

7. Pretty long and unjustified conclusion.

Reply: Due to the fact that we considered many references, mixes, the conclusion was needed to be long.

9. Many inappropriate citations.

Reply: The list of the references has been updated

10. Ill-formatted and inconsistent reference list

Reply: The list of the references has been updated
