# Supplementary material for: Evaluating the long-term strength of GGBFS-blended cement across various water-to-binder and superplasticizer ratios under heating/cooling cycles
Source: PLoS One. 2025 Jul 24;20(7):e0319923. doi: 10.1371/journal.pone.0319923 (PMC12289012; doi:10.1371/journal.pone.0319923)
Supplement: Attachment — Submitted filename: Reviewers Comments and Response.docx [file pone.0319923.s002.docx]

Dear Editor and Reviewers

Thank you very much for your help in processing the review of our manuscript. Thanks, and appreciated that you giving us the opportunity to revise our manuscript, we appreciate editor and reviewers very much for their positive and constructive comments and suggestions on our manuscript. We express our sincere gratitude to the reviewers for their time and effort dedicated to evaluating the previous version of the manuscript. Their suggestions have enabled us to improve our work. Based on the instructions provided in your letter, we uploaded the file of the revised manuscript. Accordingly, we have uploaded a copy of the original manuscript with all the major changes highlighted by *yellow color*. And some small changes and deletions have not been marked.

Appended to this letter is our point-by-point response to the comments raised by the reviewers. The comments are stated, and our responses are given directly afterward.

We would like also to thank you for allowing us to resubmit a revised copy of the manuscript.

We hope that the revised manuscript is accepted for publication in the Journal of Construction and Building Materials. The main corrections in the paper and the responses to the reviewer’s comments are as following:

**Reviewer #1: Comments**

Abstract: What is the method & conclusion?

Response: Thanks for your valuable comments the method and conclusion in the abstract have been kindly explained and improved.

There are results in Section 2 whereby it should be moved to Section 3.

Response: Thanks for your valuable comment and review. The sections are modified.

Section 3 is wee long & needs to be summarized as the current format is similar to thesis.

Response: Thanks for your valuable comments, the length of sections is due to the number of parameters. However, the entire section has been summarized and modified.

Section 5 is wee long & needs to be summarized in full sentences without bullet points.

Response: Thanks for your valuable comments, kindly that section 5 is entirely summarized and modified.

1. Please follow the format of the references.

2. There are a number of incomplete particulars such as page numbers & etc in journals.

Response: Thanks for the valuable comments. Kindly the reference style is Vancouver based on journal guidelines. And all references checked.

**Reviewer #2: Comments**

The first sentence of the abstract can be omitted

Response: Thanks for your valuable comment. Kindly, the first sentence of the abstract removed.

The first paragraph of the introduction part is not necessary. The authors can reduce it or can replace it with the physicochemical characteristics of their parent material GGBFS.

Response: Thanks for your valuable comment and review. Kindly the first paragraph removed and the suggested sentence has been added with extra modifications.

30 cyclic heating and cooling processes will be used in the study to test various temperatures in order to incorporate ongoing challenges present in the real world" Suggest some reference to this sentence

Response: Thanks for your valuable comments. Kindly, the recent references are cited and justified.

The rest part of the introduction is ok

Response: Thanks for your kind review of the entire manuscript and checked. The introduction section was modified based on your review.

Section 2.1 Mention the manufacturing details (if any) of all the materials used

Response: Thanks for your valuable comments. Kindly the manufactured detail has been added into the mentioned section.

Ground Granulated Blast Furnace Slag (GGBFS) with a specific gravity of 2.90, the bulk

density is 1000-1100 kg/m3 and the fineness is 360 more." states the source of this claim

Response: Thanks for your valuable comments. The references added and the same GGBFS manufactured by the exact company are available in the material data sheet.

Section 2.5: Authors must write one sentence about the sole purpose of this experimentation. What they wanted to achieve and how would the result help them with their objectives?

Response: Thanks for your valuable comment. Kindly, the objective of heating and cooling explained and the purpose of the experimental study.

Superscripts are missing in some places such as kg/m3, try to correct those in the revised manuscript

Response: Thanks for your valuable comment. The entire manuscript was checked and all missing superscripts were corrected.

Section 3.1: "The maximum flowability was obtained with 0.48 w/b, 2% SP, and 45% GGBFS. It can be shown that higher GGBFS replacement content led to higher flowability in all mixes" Authors can compare this result with existing literature values

Response: Thanks for your valuable comments. Kindly the result of maximum flowability has been justified.

Fig. 12c: Explain why a quadratic regression is necessary, anyways it gives a poor correlation

Response: Quadratic regression in necessary. It is the relationship between the independent and dependent variables. The poor correlation suggests that the model may be too simple to fully capture the complexity of the material interaction.

Fig. 18: EDX/FTIR/XRD data would be more beneficial

Response: Thanks for your valuable comment and kind review and I appreciate it. regarding the microstructure tests (EDX/FTIR/XRD) they are indeed crucial for thorough analysis. This advanced test is limited in my country and hard to find. I appreciate your understanding.

This section doesn't enhance the objective of the study. The authors can distribute parts of this section into the introduction, results, and conclusion.

Response: Thanks for your valuable comments. Kindly, the sustainable development section has shifted into the introduction section

Sustainable development is a separate study and many statical parameters are required to be analyzed. The authors may think of doing a separate study.

Response: Thanks for your valuable comments. Kindly the section on sustainable development was removed and the important notice has shifted to the introduction and other sections. Kindly the statistical study that ANOVA has removed, and the author thinks to do a different statical study based on your kind review. there is only remain part (prediction and actual) result.

Overall comment:

The authors must compare their results with the existing literature in form of a table. That will help the readers point out their current scenario.

Response: Thanks for your valuable comments and I appreciated it. Kindly the result of the research study justified in form of table for experimental study and comparison with literature justified through paragraph.
