# Supplementary material for: Evaluating the long-term strength of GGBFS-blended cement across various water-to-binder and superplasticizer ratios under heating/cooling cycles
Source: PLoS One. 2025 Jul 24;20(7):e0319923. doi: 10.1371/journal.pone.0319923 (PMC12289012; doi:10.1371/journal.pone.0319923)
Supplement: Attachment — Submitted filename: Response Reviewers Comments.docx [file pone.0319923.s003.docx]

Reviewer #3: Major Comments

Comment #1

Title:

The title is overly long and lacks clarity. Consider simplifying it while maintaining focus on the main research objectives.

Response: Thanks for your valuable comments. Kindly the title of research modified and reduced the length.

Comment #2
Introduction:

While the introduction highlights the importance of GGBFS, the literature review needs better integration of recent studies. Several references are outdated and lack representation of the latest advancements post-2021.

Response: Thanks for your valuable comments. Numerous research studies regarding GGBSF replacement to cement performance have been conducted and explained in detail with the latest research studies.

Comment #3
The research gap is vaguely defined. Strengthen the justification for the study by identifying more precise gaps in existing literature.

Response: Thanks for your valuable comments, The research gaps have been kindly explained. The future perspective has been added and combined with the Research gap paragraph.

Comment #4

Figures and Tables:

Figures 12a-12c and others lack clarity and are poorly illustrated. Improve their resolution, consistency in formatting, and ensure they convey the intended data trends clearly.
Tables contain significant data but need clearer captions. Ensure alignment with journal standards for table formatting.

Response: Thanks for your valuable comments. Kindly, Figure 12 has been entirely modified and the resolution of the figures has improved

Comment #5

Language:

The manuscript contains several grammatical errors and typographical issues. A professional language editing service is highly recommended to improve readability.

Response: Thanks for your valuable comments. All the grammatical errors corrected and entire manuscript checked for any typo issues and structure of sentences have been improved.

Comment #6
Conclusion:

The conclusions section is overly lengthy and repetitive. Focus on key takeaways and implications of the findings for the construction industry and sustainability.

Response: Thanks for your valuable comments, the conclusion section has been entirely improved. And the key points of finding have been explained.

Comment #7

Some acronyms, such as SP and w/b, are not defined consistently throughout the manuscript. Ensure these are explained upon first usage.
Highlight the practical implications of using GGBFS in different environmental and structural settings.
The limitations of the study are not discussed adequately. Address sample size, generalizability, and experimental constraints.

Response: Thanks for your valuable comments, The acronyms have been explained for the first use of explanation from abstract and Introduction. And regarding GGBFS a numerous of explanation of GGBFS affect included performance on different environments have kindly explained.
